# Energy Levels of Neutral Platinum

**DOI:** 10.6028/jres.097.003

**Published:** 1992

**Authors:** Jean Blaise, Jean Vergès, Jean-François Wyart, Rolf Engleman

**Affiliations:** Laboratoire Aimè Cotton,^1^ Bât. 505, C.N.R.S. II, Centre Universitaire, F-91405-ORSAY ( France); Department of Chemistry, University of New Mexico, Albuquerque, NM 87112

**Keywords:** atomic spectroscopy, electronic configurations, energy levels, platinum

## Abstract

All known energy levels of neutral platinum (Pt I) are presented, including 119 new levels based on analysis of recent comprehensive observations of the spectrum. These results are taken from a detailed analysis of the spectrum to be published in Journal de Physique II.

## 1. Introduction

An extensive analysis of the energy levels of neutral platinum (Pt I) based on new spectra recorded at the National Institute of Standards and Technology [1], at Kitt Peak National Observatory [2], and at Laboratoire Aimé Cotton has recently been prepared for publication elsewhere [3]. For completeness of the present special issue of the Journal of Research of the National Institute of Standards and Technology we list in Tables 1 and 2 the values of the Pt I energy levels. Full details of the analysis are given in Ref. [3].

## Figures and Tables

**Table 1:** Even energy levels of Ft I. The leading components of the eigenfunctions are derived from theoretical studies of the configuration groups (5*d* + 6*s*)^10^, 5*d*^9^6*d*, 5*d*^9^7*s*, 5*d*^9^7*d*, and 5*d*^9^8*s*. Where other configurations are indicated, the designations are empirical

Energy (cm^−1^)	*J*	*Leading component*	Energy (cm^−1^)	*J*	Leading component
0.	3	5*d*^9^6*s* ^3^D	63922.22	3	
775.892	2	5*d*^9^6*s* ^1^D	64128.722	5,4	
823.678	4	5*d*^8^6*s*^2 3^F	64141.155	6	5*d*^8^6*s*6*d* (^3^F_4_,^3^D)
6140.180	0	5*d*^10 1^S	64182.29	2	
6567.461	2	5*d*^9^6*s* ^3^D	64222.379	7	5*d*^8^6*s*6*d* (^3^F_4_,^3^D_3_)
10116.729	3	5*d^8^*6*s*^2I3^F	64267.43	5	5*d*^8^6*s*6*d* (^3^F_4_,^3^D)
10131.887	1	5*d*^9^6*s*^3^D	64312.78	4	5*d*^8^6*s*6*d* (^3^F_4_,^3^D)
13496.271	2	5*d*^9^6*s* ^1^D	64330.53	6	5*d*^8^6*s*6*d* (^3^F_4_)
15501.845	2	5*d*^8^6*s*^2 3^F	64379.155	5	5*d* ^8^6*s*6*d* (^3^F_4_)
16983.492	0	5*d*^8^6*s*^2 3^P	64505.839	3	5*d*^8^6*s*7s (^3^F_3_,^3^S_1_)
18566.558	1	5*d*^8^6*s*^2 3^P	64668.46	4	5*d*^8^6*s*6*d* (^3^F_4_,^3^D)
21967.111	4	5*d*^8^6*s*^2 1^G	65132.91	2	5*d*^9^7*d* ^3^P
26638.591	2	5*d*^8^6*s*^2 1^D	65308.53	4	5*d*^9^7*d* ^3^G
52379.375	3	5*d*^9^7*s* ^3^D	65339.93	5	5*d*^9^7*d* ^3^G
52667.213	2	5*d*^9^7*s* ^1^D	65346.52	3	5*d*^9^7*d* ^3^F
55640.623	5	5*d*^8^6*s*7*s* (^3^F_4_,^3^S_1_)	65361.63	1	5*d*^9^7*d* ^1^P
56784.325	4	5*d*^8^6*s*7*s* (^3^F_4_,^3^S_1_)	65381.38	4	5*d*^9^7*d* ^3^F
59591.82	1	5*d*^9^6*d* ^3^S	65387.03	3	5*d*^9^7*d* ^3^D
59731.571	2	5*d*^9^6*d* ^3^P	65395.72	2	5*d*^9^7d ^1^D
59751.177	4	5*d*^9^6*d* ^3^G	66967.965	5	5*d*^8^6*s*8*s* (^3^F_4_,^3^S_1_)
59764.266	3	5*d*^9^6*d* ^3^D	67342.66	4	5*d*^8^6*s*8*s* (^3^F_4_,^3^S_1_)
59782.853	1	5*d*^9^6*d* ^1^P	68006.95	3	5*d*^9^6*d* ^3^G
59812.72	5	5*d*^9^6*d* ^3^G	68072.245	3	5*d*^9^6*d* ^3^F
59872.140	3	5*d* ^9^6*d* ^1^F	68094.74	2	5*d*^9^6*d* ^3^F
59882.421	4	5*d*^9^6*d* ^3^F	68121.56	4	5*d*^9^6*d* ^3^G
59908.170	2	5*d*^9^6*d* ^1^D	68169.42	2	5*d*^9^6*d* ^1^D
60357.804	1	5*d*^9^7*s* ^3^D	68275.31	2	
60573.69	0	5*d*^9^6*d* ^1^S	68703.45	4	
60640.669	2	5*d*^9^7*s* ^3^D	68716.32	6	5*d* ^8^6*s*6*d* (^3^F_4_,^1^D_2_)
60790.393	3	5*d*^8^6*s*7*s* (^3^F_4_,^3^S_1_)	68759.01	4	
60884.001	4	5*d*^8^6*s*7*s* (^3^F_4_,^1^S_0_)	68831.115	5	
62567.995	3	5*d*^9^8*s* ^3^D	68912.21	4	
62705.33	2	5*d*^9^8*s* ^1^D	68947.47	3	

**Table 2:** Odd energy levels of Pt I. The leading components of the eigenfunctions are derived from theoretical studies of the mixed group of configurations 5*d*^9^6*p +*5*d*^8^6*s*6*p +*5*d*^7^6*s*^2^6p + 5*d*^9^7*p*. Where other configurations are indicated, the designations are empirical

Energy (cm^−1^)	*J*	Leading component	Energy (cm^−1^)	*J*	Leading component
30156.854	4	5*d*^8^6*s*6*p* (^4^F)^5^D	54839.206	3	5*d*^8^6*s*6*p* (^4^P)^3^D
32620.018	2	5*d*^9^6*p* ^3^P	55009.37	4	5*d*^8^6*s*6*p* (^2^G)^3^H
33680.402	5	5*d*^8^6*s*6*p* (^4^F)^5^F	55216.828	1	5*d*^9^6*p* ^3^D
34122.165	3	5*d*^9^6*p* ^3^F	55536.276	3	5*d*^8^6*s*6*p* (^2^P)^3^D
35321.653	3	5*d*^8^6*s*6*p* (^4^F)^5^D	55984.51	5	5*d*^8^6*s*6*p* (^2^G)^3^H
36296.310	4	5*d*^8^6*s*6*p* (^4^F)^5^G	56288.65	4	5*d*^8^6*s*6*p* (^2^G)^3^F
36781.551	6	5*d*^8^6*s*6*p* (^4^F)^5^G	56670.20	2	5*d*^8^6*s*6*p* (^4^P)^3^P
36844.710	1	5*d*^9^6*p* ^3^P	56794.43	5	5*d*^7^6*s*^2^6*p* ^4^F^*3^G
37342.101	2	5*d*^9^6*p* ^3^P	57041.73	1	5*d*^8^6*s*6*p* (^4^P)^3^P
37590.569	4	5*d*^9^6*p*^3^F	57506.187	3	5*d*^8^6*s*6*p* (^2^G)^3^F
37769.073	3	5*d*^9^6*p* ^3^D	57987.392	2	5*d*^9^7*p* ^3^P
38536.160	5	5*d*^8^6*s*6*p* (^4^F)^5^F	58101.17	3	5*d*^9^7*p* ^3^F
38815.908	2	5*d*^8^6*s*6*p* (^2^D)^3^F	58326.75	2	5*d*^8^6*s*6*p* (^2^P)^3^D
40194.228	4	5*d*^8^6*s*6*p* (^4^F)^5^F	58388.47	4	5*d*^7^6*s*^2^6*p* ^4^F^*5^G
40516.243	2	5*d*^8^6*s*6*p* (^4^F)^5^D	58482.14	3	5*d*^9^*p* ^1^F
40787.857	2	5*d*^8^6*s*6*p* (^4^P)^5^P	58780.80	1	5*d*^9^7*p* ^1^P
40873.529	0	5*d*^8^6*s*6*p* (^2^D)^3^P	59127.72	2	5*d*^8^6*s*6*p* (^2^D)^3^F
40970.165	3	5*d*^8^6*s*6*p* (^4^F)^5^G	59346.33	4	5*d*^9^7*p* ^3^F
41802.744	1	5*d*^8^6*s*6*p* (^2^D)^3^D	59462.28	2	5*d*^9^7*p* ^1^D
42660.058	3	5*d*^8^6*s*6*p* (^4^F)^5^D	59492.41	4	5*d*^9^7*p* ^3^F
43187.836	1	5*d*^8^6*s*6*p* (^4^F)^5^D	59686.20	3	5*d*^7^6*s*^2^6*p* ^4^F^* 5^G
43945.543	3	5*d*^8^6*s*6*p* (^4^P)^5^P	5979Z23	1	5*d*^8^6*s*6*p* (^4^P)^3^S
44432.663	4	5*d*^8^6*s*6*p* (^4^F)^5^G	59916.97	2	5*d*^9^7*p* ^1^D
44444.364	2	5*d*^8^6*s*6*p* (^4^F)^5^F	59920.03	3	5*d*^9^7*p* ^3^D
44730.313	3	5*d*^8^6*s*6*p* (^2^F)^3^D	60328.02	3	5*d*^8^6*s*6*p* (^4^F)^3^F
45398.478	1	5*d*^8^6*s*6*p* (^4^P)^5^P	60423.93	4	5*d*^8^6*s*6*p* (^2^G)^3^G
46170.386	2	5*d*^9^6*p* ^3^F	60441.30	1	5*d*^9^7*p* ^1^P
46419.962	2	5*d*^8^6*s*6*p* (^4^P)^5^D	61097.48	2	5*d*^8^6*s*6*p* (^2^G)^3^F
46433.912	0	5*d*^8^6*s*6*p* (^4^P)^5^D	61352.25	3	5*d*^8^6*s*6*p* (^2^G)^3^F
46622.489	3	5*d*^8^6*s*6*p* (^2^F)^3^D	61633.79	5	5*d*^8^6*s*6*p* (^2^G)^3^G
46792.965	5	5*d*^8^6*s*6*p* (^4^F)^3^G	61645.33	2	5*d*^8^6*s*6*p* (^2^G)^3^F
46963.670	4	5*d*^8^6*s*6*p* (^4^P)^5^D	6194222	4	5*d^8^*(^3^F_4_)6*s*7*p*(^3^P_0_)
47740.565	1	5*d*^9^6*p* ^1^P	6206229	2	5*d*^7^6*s^2^*6*p* ^4^F^*5^G
48351.94	4	5*d*^8^6*s*6*p* (^4^F)^3^F	62106.38	3	5*d*^8^6*s*6*p* (^4^P)^3^D
48535.596	2	5*d*^8^6*s*6*p* (^4^F)^5^G	62321.92	3	5*d*^8^6*s*6*p* (^2^D)^3^F
48779.337	3	5*d*86*s*6*p* (^4^F)^3^D	62510.36	4	5*d*^8^(^3^F_4_)6*_S_*7*p*(^3^P_1_)
49286.116	3	5*d*^8^6*s*6*p* (^4^P)^5^P	62659.30	2	5*d*^8^6*s*6*p* (^4^P)^3^D
49544.565	1	5*d*^9^6*p* ^3^D	62835.58	5	5*d*^8^(^3^F_4_)6*s*7*p*(^3^P_1_)
49880.883	2	5*d*^9^6*p* ^3^D	63067.47	1	5*d*^8^6*s*6*p* (^2^D)^3^D
50010.155	4	5*d*^8^6*s*6*p* (^2^F)^3^F	63167.33	3	5*d*^8^(^3^F_4_)6*s*7*p*(^3^P_1_)
50055.313	1	5*d*^8^6*s*6*p* (^4^F)^5^F	6335291	6	5*d*^8^(^3^F_4_)6*s*7*p*(^3^P_2_)
50299.385	5	5*d*^7^6*s*^2^6*p* ^4^F^*3^G	63466.29	1	5*d*^8^6*s*6*p* (^2^P)^3^P
50387.66	0	5*d*^9^6*p* ^3^P	63826.31	2	5*d*^7^6*s^2^*6*p* ^4^F^*5^G
51097.529	3	5*d*^8^6*s*6*p* (^4^P)^5^D	63945.05	5	5*d*^8^(^3^F_4_)6*s*7*p*(^3^P_2_)
51286.946	2	5*d*^8^6*s*6*p* (^2^F)^1^D	64248.95	2	5*d*^8^6*s*6*p* (^2^D)^3^D
51545.544	3	5*d*^8^6*s*6*p* (^4^F)^3^D	64319.385	4	5*d*^8^(^3^F_4_)6*s*7*p*(^3^P_2_)
51753.317	2	5*d*^8^6*s*6*p* (^2^F)^3^F	64515.68	2	5*d*^7^6*s*^2^6*p* ^4^F^*5^G
52071.684	1	5*d*^8^6*s*6*p* (^4^P)^5^D	64619.64	1	5*d*^8^6*s*6*p* (^4^P)^3^D
52438.59	5	5*d*^8^6*s*6*p* (^2^F)^3^G	64675.92	3	5*d*^7^6*s*^2^6*p* ^4^P^*5^D
52520.13	4	5*d*^8^6*s*6*p* (^2^F)^3^F	64904.25	3	5*d*^8^(^3^F_4_)6*s*7*p*(^3^P_2_)
52708.365	2	5*d*^8^6*s*6*p* (^4^P)^5^D	65306.80	1	5*d*^9^5*f*
53019.303	1	5*d*^8^6*s*6*p* (^2^F)^3^D	65315.89	2	5*d*^9^5*f*
53665.25	1	5*d*^8^6*s*6*p* (_2_P)^3^D	65318.95	6	5*d*^9^5*f*
53953.379	2	5*d^8^*6*s*6*p* (^2^P)^3^P	65325.49	2	5*d*^9^5*f*
54011.150	3	5*d*^8^6*s*6*p* (^4^P)^5^P	65331.20	3	5*d*^9^5*f*
54133.26	2	5*d*^8^6*s*6*p* (^4^P)^5^S	6533243	1	5*d*^9^5*f*
54178.47	4	5*d*^8^6*s*6*p* (^4^P)^5^D	65333.25	4	5*d*^9^5*f*
65336.49	3	5*d*^9^5*f*	67303.64	3,4	5*d*^8^6*s*7*p*
65339.66	4	5*d*^9^5*f*	67413.65	5,4	5*d*^8^6*s*7*p*
65341.92	5	5*d*^9^5*f*	68266.90	5	5*d*^8^6*s*7*p*
65510.22	3		68343.55	3,4	5*d*^8^6*s*7*p*
65697.70	2,1		68606.62	2	
65850.11	1		68657.42	3	
65852.56	4		70087.93	7	5*d*^8^(^3^F_4_)6*s*5*f*
66198.85	2		70088.64	5,6	5*d*^8^(^3^F_4_)6*s*5*f*
6643156	1		70095.52	6	5*d*^8^(^3^F_4_)6*s*5*f*
66927.43	2	5*d*^9^7*p*(^2^D_3/2_,^2^P_1/2_)	70099.57	5	5*d*^8^(^3^F_4_)6*s*5*f*
67121.58	3	5*d*^9^7*p*(^2^D_3/2_,^2^P_3/2_)			
